# Electronic structure of superconducting VN(111) films

**DOI:** 10.1186/s11671-024-03978-x

**Published:** 2024-03-11

**Authors:** Rongjing Zhai, Jiachang Bi, Shun Zheng, Wei Chen, Yu Lin, Shaozhu Xiao, Yanwei Cao

**Affiliations:** 1 Ningbo Institute of Materials Technology and Engineering, Chinese Academy of Sciences, Ningbo, 315201 China; 2 University of Science and Technology of China, Hefei, 230026 China; 3 State Key Laboratory of Functional Materials for Informatics, Shanghai Institute of Microsystem and Information Technology, Chinese Academy of Sciences, Shanghai, 200050 China; 4 Hefei Innovation Research Institute, Beihang University, Hefei, 230013 China; 5 Yongjiang Laboratory, Ningbo, 315202 Zhejiang China; 6 Center of Materials Science and Optoelectronics Engineering, University of Chinese Academy of Sciences, Beijing, 100049 China

**Keywords:** Vanadium nitride, Epitaxy, Superconductivity, Electronic structure

## Abstract

Vanadium nitride (VN) is a transition-metal nitride with remarkable properties that have prompted extensive experimental and theoretical investigations in recent years. However, there is a current paucity of experimental research investigating the temperature-dependent electronic structure of single-crystalline VN. In this study, high-quality VN(111) films were successfully synthesized on $\alpha$-Al_2_O_3_(0001) substrates using magnetron sputtering. The crystal and electronic structures of the VN films were characterized by a combination of high-resolution X-ray diffraction, low-energy electron diffraction, resonant soft X-ray absorption spectroscopy, and ultraviolet photoelectron spectroscopy. The electrical transport measurements indicate that the superconducting critical temperature of the VN films is around 8.1 K. Intriguingly, the temperature-dependent photoelectron spectroscopy measurements demonstrate a weak temperature dependence in the electronic structure of the VN films, which is significant for understanding the ground state of VN compounds.

## Introduction

Due to the emergence of various extraordinary properties, the study of vanadium-based compounds, such as VO_2_, VSe_2_, SrVO_3_, CsV_3_Sb_5_, have recently garnered significant attention [1–4]. Among these compounds, vanadium nitride (VN) exhibits an excellent combination of high thermal and chemical stability, superconductivity, and high hardness, making it widely applicable in multifunctional coatings, catalysis, and supercapacitors [5–7]. More interestingly, VN films can be ideal parent compounds to prepare high-quality and superior-performing VO_2_ films [8]. To comprehensively understand the intrinsic properties of VN compounds, it is necessary to synthesize single-crystalline VN films and conduct high-resolution measurements of their electronic structure.

With the advancements in film deposition techniques, single-crystalline VN films have been successfully synthesized using pulsed laser deposition and magnetron sputtering methods [9, 10]. Therefore, it is feasible to experimentally elucidate various intrinsic optical and thermal properties of these films, including the complex dielectric functions, the tetragonal-to-cubic structural phase transition, and the thermal conductivity [10–12]. However, the electric transport measurements of previous (111)-oriented VN thin films on $\alpha$-Al_2_O_3_(0001) substrates were only conducted down to a temperature of 10 K [9]. As a result, it remains uncertain whether superconductivity is present in these films or not. Additionally, there are currently no reports on the temperature-dependent electronic structure of VN.

To tackle the aforementioned issue, high-quality VN(111) epitaxial films were synthesized on (0001)-Al_2_O_3_ single crystal substrates ($5\times 5 \times 0.5$ mm^3^) by high-pressure magnetron sputtering. The crystal and electronic structures of VN films were characterized by employing a combination of high-resolution X-ray diffraction (XRD), low-energy electron diffraction (LEED), resonant soft X-ray absorption spectroscopy (XAS), and ultraviolet photoelectron spectroscopy (UPS). The electrical transport measurements reveal that the superconducting critical temperature of the VN films is near 8.1 K.

**Fig. 1:**
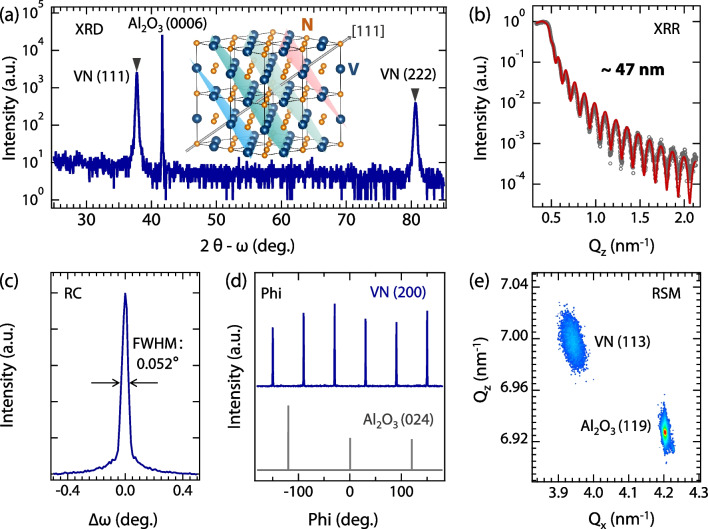
Crystal structure of VN films. **a** Wide-range 2$\theta$-$\omega$ scan of VN/Al_2_O_3_(0001). The black triangles indicate the (111) and (222) peaks of VN films. The inset is the schematic of the VN crystal structure. **b** XRR (dots) and the fitted curves (solid line) of VN films. **c** Rocking curve recorded around VN(111) diffractions. **d** Phi scans of VN/Al_2_O_3_(0001) around VN (200) diffraction and Al_2_O_3_ (024) diffraction. **e** RSM patterns of VN/Al_2_O_3_(0001) around Al_2_O_3_ (119) diffraction and VN (113) diffraction

## Results and discussion

**Fig. 2:**
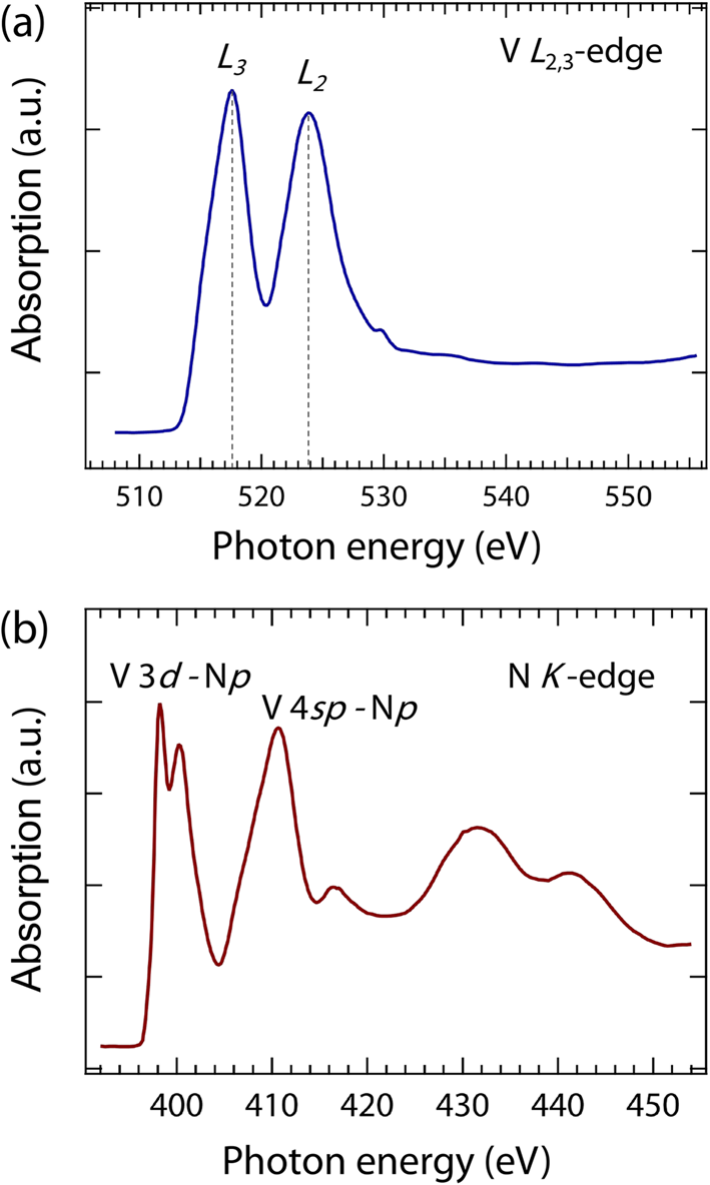
XAS at **a** V $L_{2,3}$-edge, and **b** N *K*-edge on VN film with a total electron yield (TEY) detection mode at room temperature

**Fig. 3:**
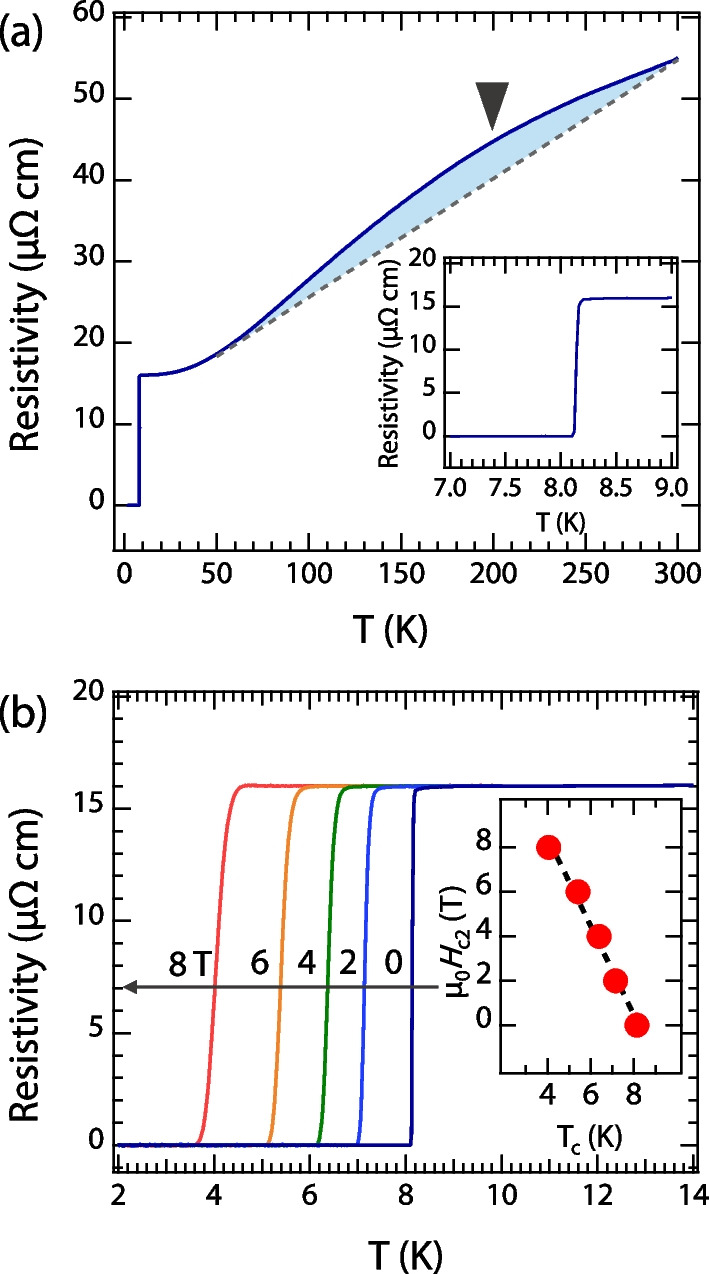
Transport properties of VN film. **a** Resistivity vs temperature $\rho (T)$ curves from 1.8 to 300 K. The insert is the extended view of temperature-dependent resistivity of VN films near $\text {T}_\text {c}$. **b** Resistivity of VN films as a function of temperature under perpendicular magnetic fields from 0 to 8 T. The inset shows the upper critical field as a function of $\text {T}_\text {c}$, derived from the data in **b**

**Fig. 4:**
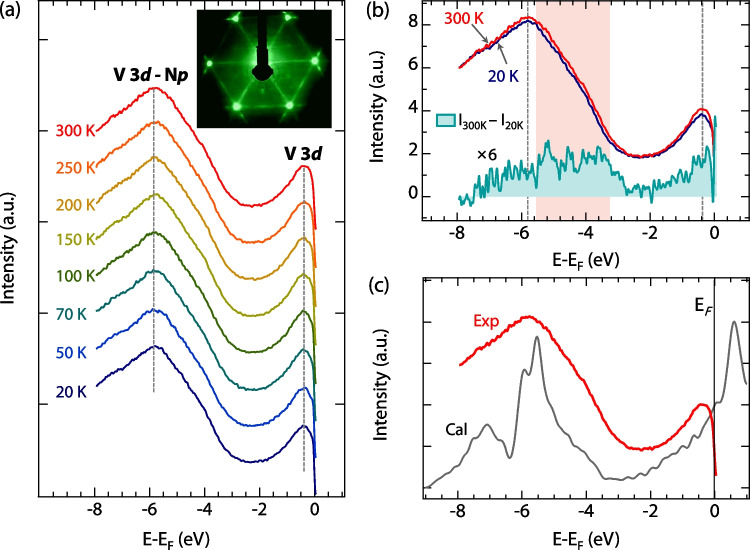
**a** Temperature-dependent valence-band spectra of VN films, the inset is the LEED pattern of VN(111) films with the electron energy at 100 eV. **b** The difference of the valence-band spectra of VN films measured at 20 (the blue curve) and 300 K (the red curve). **c** The UPS data (red line) and the density of states (black line) of VN films

Initially, XRD was conducted to characterize the crystal structure of the VN films. The 2$\theta$-$\omega$ scan in Fig. 1a shows only (111) and (222) diffraction peaks of the VN film without any detectable secondary phases. Furthermore, the averaged grain size of VN films ($\sim$ 41 nm) can be estimated by the Scherrer equation [13, 14]. The thickness ($\sim$ 47 nm, as seen in Fig. 1b) of the films was estimated by fitting the XRR data with a slab-model approach in REFLEX software, which incorporates the Abeles matrix method to account for the thickness of films [15–18]. The distinct thickness fringes observed in the X-ray reflection (see Fig. 1b) and the narrow rocking curves ($\sim$ 0.052^∘^, as seen in Fig. 1c) of the VN films provide confirmation of their smooth surface morphology (with a roughness $\sim$ 0.3 nm) and high-quality crystalline structure. The XRR detection employs a parallel beam, and the X-ray spot can fully cover the samples (the surface area of the films is $5\times 5$ mm^2^). The in-plane crystal structure of the VN films was determined using Phi scans. As displayed in Fig. 1d, the VN films exhibit a six-fold symmetry with an azimuthal rotation of 30^∘^ relative to $\alpha$-Al_2_O_3_(0001) substrates, which is consistent with the crystal orientation of TiN films on $\alpha$-Al_2_O_3_(0001) substrates [19]. Figure  1e shows the reciprocal space mapping (RSM) patterns of VN/Al_2_O_3_(0001) around Al_2_O_3_(119) diffraction and VN (113) diffraction. The (111) and (11$\bar{2}$) layer spacings extracted from the RSM are $\sim$2.382 Å  and $\sim$1.693 Å, respectively. The corresponding lattice parameters are *a* = 4.147 Å and *c* = 4.126 Å, which is very close to the value of the bulk VN (4.132 Å), indicating the epitaxy of VN films.

Next, we investigate the electronic band structure of unoccupied states in VN films by XAS at the V $L_{2,3}$- and N *K*-edges. The measurements were performed at room temperature using a surface-sensitive total electron yield (TEY) detection mode. Figure 2a shows two distinct characteristic peaks of the V $L_{2,3}$-edge (indicated by the gray dotted lines) at $\sim$ 517.6 and 523.8 eV, which correspond to the transition of V 2*p* to V 3*d* states. The N *K*-edge of VN films (see Fig. 2b) is consistent with the previous reports [20], the peaks at around 398.2 and 400.2 eV correspond to the V 3*d*-hybridized states, while the peak at around 410.6 eV is assigned to the combination of N *p* and V 4*sp* states. Furthermore, features observed near 416.4 eV and above are attributed to N *p* states mixed with the extended set orbitals [20].

To characterize the superconductivity properties of VN films, we conducted temperature-dependent electrical transport measurements using a van der Pauw geometry. Figure 3a illustrates the reduction in resistivity of VN films as the temperature is lowered from 300 to 8.2 K, consistent with previous reports [11]. Notably, an obvious broad bump at around 200 K can be observed, which is in contrast to the behavior of TiN films that exhibit a linear relation between $\rho$ and *T* over a broad temperature [21]. This bump of temperature-dependent resistivity can be connected to the tetragonal-to-cubic structural phase transition [11]. As the temperature further decreases, a sharp transition from the normal to superconducting states occurs at 8.2 K, and zero resistance is achieved at 8.1 K. We further examine the effect of perpendicular magnetic fields on the superconducting critical temperature. Figure 3b shows the resistivity measured with variable magnetic fields. As seen, the $\text {T}_\text {c}$ is gradually suppressed by the magnetic field, the $\text {T}_\text {c}$ decreases from 8.14 to 4.05 K upon the application of an 8 T magnetic field. The inset in Fig. 3b depicts the relationship between the upper critical fields ($\mu _{0}H_{c2}$) and the transition temperatures ( $\text {T}_\text {c}$). The estimated Ginzburg-Landau superconducting coherence length $\xi _{0}$ is $\sim$ 5.4 nm at zero temperature with the formula $\xi _{0}$ [$(\xi _{0})^{2}=- \Phi _{0}/2\pi T_{c} (\mu _{0}dH_{c2}/dT\mid _{T_{c}})$ [22, 23]]

At last, valence-band spectra were investigated using UPS to determine their temperature dependence. Since the UPS is a surface-sensitive technique [24], VN films were annealed at 580 ^∘^C for 5 h before the UPS measurements to remove the surface contaminations. The insert in Fig. 4a shows a sharp LEED pattern with 1$\times$1 hexagonal diffraction spots, indicating a clean and ordered surface of the VN films. Figure 4a shows the valence-band spectra of VN films measured at temperatures ranging from 20 to 300 K, revealing two prominent peaks at approximately $-$ 0.4 and $-$ 5.8 eV [25, 26]. The observed structure between 0 and -2 eV below the Fermi level (E_F_) arises from the V 3d states, the maximum intensity is observed around $-$ 5.8 eV, which is attributed to hybridized V 3*d* and N 2*p* states [26]. It is worth noting that both the line shape and the peak positions (around $-$ 5.8 and $-$ 0.4 eV, as indicated by the guided lines in Fig. 4a) exhibit no significant changes with temperatures ranging from 20 to 300 K. Hence, the connection between the structural phase transition and the UPS data of VN is weak. To precisely compare the temperature-dependent differences in the valence band structure, the spectra measured at 300 and 20 K were selected and presented in Fig. 4b. The discrepancy between them was obtained by subtracting ${I}_{20K}$ from ${\text{I}}_{300K}$, as depicted by the cyan area in Fig. 4b. Detectable signals can be observed at the positions of the two prominent peaks, and the discrepancy also exhibits distinctive features near the shoulder of the peak at approximately $-$ 5.8 eV, as indicated by the orange areas. This distinct shoulder near the peak around $-$ 5.8 eV has been previously reported [26]. To further understand the UPS data, we also carried out DFT calculations. As seen in Fig. 4c, the main feature of the UPS data can be well reproduced by the DFT calculations, which can be assigned to the broad peak at around $-$ 5.8 eV.

## Conclusion

In summary, high-quality VN(111) films were successfully synthesized on $\alpha$-Al_2_O_3_(0001) substrates using magnetron sputtering. The crystalline and electronic structures of the VN films were characterized using a combination of high-resolution XRD, LEED, XAS, and UPS. The electrical transport measurements indicate a superconducting critical temperature of approximately 8.1 K for the VN films. The temperature-dependent photoelectron spectroscopy measurements indicate a weak dependence of the electronic structure of the VN films on temperature.

## Experiments and computation methods

Thin VN films with a thickness of nearly 47 nm were synthesized on $\alpha$-Al_2_O_3_(0001) substrates using a home-made high-pressure radio frequency (RF) magnetron sputtering system. The sputtering process employed a 2-inch V (99.995% purity) target and N_2_ (99.999% purity) as the reactive gas. The sputtering system maintained a base vacuum pressure of 3 $\times$10^-8^ Torr. During the growth process, the N_2_ pressure was maintained at 0.02 Torr with a gas flow rate of 3.2 sccm, while the substrate temperature was set at 900 ^∘^C. The sample holder rotated at a speed of 5 rpm during the growth process. The RF generator was operated at a power of 80 W. The distance between the substrate and target surface centers is 80 mm. The growth rate of VN films is $\sim$ 0.26 nm/min.

The crystal structure of VN films was characterized using a high-resolution XRD diffractometer (Bruker D8 Discover) with the Cu K_α_ source ($\lambda$ = 1.5406 Å). The electrical properties were measured utilizing a Physical Property Measurement System (from Quantum Design) with a 50 $\upmu A$ current in a van der Pauw geometry. The electronic structures of VN films were investigated by the ultraviolet photoelectron spectroscopy with a helium discharge lamp ($h\upsilon$ = 21.218 eV) and an advanced spectrometer (DA30, Scienta Omicron). The base pressure was lower than 5 $\times$ 10^-11^ mbar. The total energy resolution was higher than 10 meV.

The XAS measurements were conducted at beamline 02B02 of the SiP$\cdot$ME2 platform at the Shanghai Synchrotron Radiation Facility. The beamline delivers soft x-ray photons with photon flux around 1 $\times$ 10 ^11^ photons/s @ E/$\Delta$E = 3700 and a tightly focused beam spot size ($\sim$ 150 $\upmu \hbox {m}$ $\times$ 50 $\upmu \hbox {m}$) at the sample [27]. The base pressure of the XAS chamber was around 2 $\times$ 10^-9^ mbar. The angle between the incident light and the films is approximately 50 degrees.

The theoretical DOS of VN was obtained through DFT calculations by employing the generalized gradient approximation [28] using the projected augmented wave method [29] as implemented in the QUANTUM ESPRESSO code [30, 31]. The plane-wave kinetic cut-off energy was set to be 60 Ry, and the Brillouin zone was sampled with a **k** mesh of 24$\times$24$\times$24. During the calculations, the on-site Hubbard interaction ($\textrm{U}_\textrm{eff}$ = 4 eV) was invoked for the V 3*d* electrons, and relativistic effects, including SOC, were also taken into account.

## Data Availability

The authors declare that the data supporting the findings of this study are available in the paper. If any other format of data files is required, they are available from the corresponding author upon reasonable request.
